# Prevalence and molecular characterization of *Cryptosporidium* in giant panda (*Ailuropoda melanoleuca*) in Sichuan province, China

**DOI:** 10.1186/s13071-015-0953-8

**Published:** 2015-06-25

**Authors:** Tao Wang, Zuqin Chen, Yue Xie, Rong Hou, Qidun Wu, Xiaobing Gu, Weiming Lai, Xuerong Peng, Guangyou Yang

**Affiliations:** Department of Parasitology, College of Veterinary Medicine, Sichuan Agricultural University, No.46, Xingkang Road, Yucheng District, 611130 Chengdu, China; Chengdu Research Base of Giant Panda Breeding, 610081 Chengdu, China; Department of Chemistry, College of Life and Basic Science, Sichuan Agricultural University, 625014 Ya’an, China

**Keywords:** *Cryptosporidium andersoni*, 18SrRNA, Giant panda, Prevalence, China

## Abstract

**Background:**

*Cryptosporidium* spp. have been extensively reported to cause significant diarrheal disease in humans and domestic animals. On the contrary, little information is available on the prevalence and characterization of *Cryptosporidium* in wild animals in China, especially in giant pandas. The aim of the present study was to detect *Cryptosporidium* infections and identify *Cryptosporidium* species at the molecular level in both captive and wild giant pandas in Sichuan province, China.

**Findings:**

Using a PCR approach, we amplified and sequenced the 18S rRNA gene from 322 giant pandas fecal samples (122 from 122 captive individuals and 200 collected from four habitats) in Sichuan province, China. The *Cryptosporidium* species/genotypes were identified via a BLAST comparison against published *Cryptosporidium* sequences available in GenBank followed by phylogenetic analysis. The results revealed that both captive and wild giant pandas were infected with a single *Cryptosporidium* species, *C. andersoni*, at a prevalence of 15.6 % (19/122) and 0.5 % (1/200) in captive and wild giant pandas, respectively.

**Conclusions:**

The present study revealed the existence of *C. andersoni* in both captive and wild giant panda fecal samples for the first time, and also provided useful fundamental data for further research on the molecular epidemiology and control of *Cryptosporidium* infection in giant pandas.

## Findings

### Background

*Cryptosporidium* spp. are protozoan parasites that cause significant gastrointestinal disease in humans, domestic animals and wild vertebrates [1, 2]. Hosts get infected via the oral ingestion of oocysts that are excreted with feces by infected hosts [3]. Currently, delimiting species within the genus *Cryptosporidium* has also been controversial, but at least 27 *Cryptosporidium* species have been recognized as valid [4]. Of these, 14 species (over 30 *Cryptosporidium* genotypes) were known to infect wild animals, such as bears, lesser panda, raccoons, beavers, kangaroos, squirrels, monkeys, minks, mongooses, tortoise and finches [5–7].

The giant panda (*Ailuropoda melanoleuca*) is one of the world’s most recognized and threatened wild animals with an estimated wild population size of only 1600. These individuals are restricted to six isolated mountain ranges in China, *i.e.* Minshan, Qionglai, Qinling, Daxiangling, Xiaoxiangling and Liangshan mountains. More precisely, they are mainly distributed in two of them --- Minshan (44.4 %) and Qiongla mountains (27.4 %) of Sichuan province [8]. To protect this endangered and valuable animal, several giant panda research bases, conservation centers and zoos have been established [8], raising 394 captive giant pandas, mostly in Sichuan province (85 %). Compared to the extensive studies on *Cryptosporidium* infection in humans and domestic animals hosts, fewer investigations have been conducted in wild animal species, especially in giant pandas. To date, only one *Cryptosporidium* infection case has been reported in an 18-year-old male captive giant panda in Sichuan province, China [9]. Moreover, there have been no large-scale *Cryptosporidium* prevalence studies of either wild or captive pandas in China. In order to gain more insight into the *Cryptosporidium* infection in giant pandas, we used molecular diagnostic tools to detect and characterize *Cryptosporidium* in both wild and captive pandas (approximately 31 % of the captive population) in Sichuan province, China.

### Methods

#### Ethics statement

Samples were collected after defecation under the permission of the relevant institutions. All procedures were reviewed and approved by the Wildlife Management and Animal Welfare Committee of China. During fecal collection, animal welfare was taken into consideration.

#### Fecal sample collection

Captive giant panda fecal samples (n = 122, each from one single animal) were obtained from two panda conservation centers (CRB: Chengdu Research Base of Giant Panda; CCRC: China Conservation and Research Centre for the Giant Panda) in Sichuan province, China. Fecal samples (n = 200) from wild giant pandas were collected from four panda’s mountain habitats in Sichuan province during the 4th national investigation of giant pandas (Table 1). The samples were labeled and placed immediately in disposable plastic bags before being shipped to the laboratory of Sichuan Agricultural University for purification and processing.

**Table 1:** Prevalence of *Cryptosporidium* infection in captive and wild giant pandas in Sichuan province, China

	Location	No. of samples collected	No. of samples positive	% positive
Captive				
	CRB	55	2	3.6
	CCRC	67	17	25.4
	Total	122	19	15.6
Wild				
	Daxiangling mountains	12	0	0
	Liangshan mountains	16	0	0
	Minshan mountains	145	1	0.7
	Qionglai mountains	27	0	0
	Total	200	1	0.5

#### Sample processing and DNA extraction

Oocysts were concentrated from feces as previously described [10]. Briefly, each fecal sample was mixed with 15 ml of dH_2_O in specimen cups. The suspension was then transferred to another clean specimen cup through a sieve with an 80 mm pore size to remove larger fecal debris. The filtrate was then centrifuged at 1800 *× g* for 15 min and the supernatant was removed. Total DNA was extracted from 200 mg of each oocyst precipitation using a QIAamp DNA Mini Stool Kit (Qiagen, Hilden, Germany) according to the manufacturer’s protocol. The eluted DNA was stored at -20 °C prior to PCR analysis. DNA from *Cryptosporidium* obtained from a giant panda [9], kindly provided by the key laboratory of Animal Disease and Human Health of Sichuan Province, College of Veterinary Medicine, Sichuan Agricultural University, was used as a positive control, and deionized water was used as a negative control.

#### Gene amplification and sequencing

A PCR amplification approach was used to detect *Cryptosporidium* in DNA samples. We amplified a fragment (~830 bp) of the 18S ribosomal RNA (18S rRNA) gene as described previously [11]. During the PCR, the primer pairs (forward: 5′-TTCTAGAGCTAATACATGCG-3′ and reverse: 5′-CCCATTTCCTTCGAAACAGGA-3′) were used. The PCR mixture contained 12.5 μl Taq PCR MasterMix (Tiangen Biochemical Technology Co., Ltd.), 0.5 μl BSA (0.1 g/10 ml), 8 μL ddH_2_O, 2 μL DNA and 1 μL of each forward and reverse primer (working concentration: 10pmol/L) in a 25 μl reaction volume. Each of the 35 PCR cycles consisted of 94 °C for 45 s, 59 °C for 45 s, and 72 °C for 1 min after an initial hot start at 94 °C for 3 min and ending with 72 °C for 7 min. PCR products were analyzed on a 1 % agarose gel and stained with GoldenView for visualisation. Because of the weakness of gel bands of interest, all the PCR products were purified and cloned into a pMD19-T vector before sent for commercial sequencing (Invitrogen, Shanghai).

#### Phylogenetic analysis

The obtained sequences were aligned with each other and published 18S rRNA gene sequences of *Cryptosporidium* spp. using the software ClustalX (http://www.clustal.org) to determine *Cryptosporidium* species. Phylogenetic relationships of *Cryptosporidium* spp. were reconstructed using the Neighbour-Joining (1000 replicates) analysis implemented in MEGA 5.0 (http://www.megasoftware.net/) based on genetic distances calculated by the Kimura 2-parameter model. All the positive sequences (n = 20) generated from PCR analysis of the ~830 bp fragment of the 18S rRNA gene were used in the phylogenetic analysis. *Eimeria tenella* (GenBank accession number: U40264) was used as the out-group.

#### Statistical analysis

Statistical analysis was performed using KF software (chi-square test, 1.0).

### Results

A total of 322 fecal samples (122 captive ones and 200 wild ones) were screened for the presence of *Cryptosporidium* by PCR amplification of the 18S rRNA gene. Of 122 fecal samples of captive pandas collected from conservation centers, 19 (15.6 %) were positive for *Cryptosporidium* infection (Table 1). *Cryptosporidium* was found across all gender and age groups (juvenile panda: 1.5–5.5 years, adult panda: >5.5 years [8]) of captive pandas (Table 2). Data showed that 21.9 % (15/73) of females and 14.3 % (4/49) of males were positive for *Cryptosporidium*, whilst 13.6 % (2/22) juvenile pandas and 17.0 % (17/100) adult pandas were identified as *Cryptosporidium* positive by PCR. However, these differences were not significant (different gender: *P* value = 3.45; different age: *P* value = 0.36).

**Table 2:** Prevalence of *Cryptosporidium* infection in captive giant pandas in Sichuan province, China by gender and age group

	Gender group		Age group	
	Female	Male	Juvenile (1.5–5.5 years)	Adult (>5.5 years)
No. of samples collected	73	49	22	100
No. of samples positive	15	4	2	17
% positive	20.1	8.2	9.1	17.0

Only one (0.5 %) sample from Minshan mountains was detected as *Cryptosporidium* positive out of all 200 fecal samples of wild pandas in Sichuan province (Table 1).

**Fig. 1:**
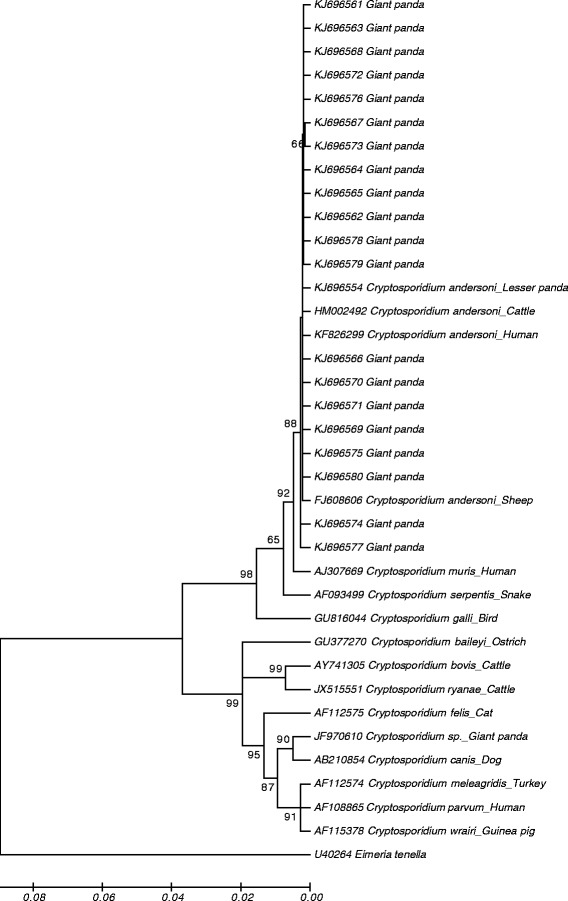
Phylogenetic analysis of *Cryptosporidium* spp. using Neighbor Joining method based on sequences of 18S rRNA genes. The numbers at clades indicate bootstrap values

According to obtained phylogenetic tree (Fig. 1), the *Cryptosporidium* isolates analyzed in this study cluster together with the strain described as *C. andersoni* (with 94.1–99.8 % identity) genotype originated from cattle. The nucleotide sequences of *Cryptosporidium* from giant pandas in this study were deposited in GenBank under accession numbers KJ696561 to KJ696580.

### Discussion

Consistent with the previous study by Liu *et al.* [9], our results confirmed the presence of *Cryptosporidium* infection in captive giant pandas. Moreover, our findings demonstrated for the first time the presence of *Cryptosporidium* in wild giant panda fecal samples. Interestingly, phylogenetic analysis based on 18S rRNA gene, indicated that the *Cryptosporidium* isolate analyzed in this study clusters together with the strain described as *C. andersoni* (with 94.1–99.8 % identity) genotype originated from cattle, rather than recently reported genotype: the *Cryptosporidium* giant panda genotype (with 84.6–89.8 % identity). Notably, a distinct *Cryptosporidium* genotype and a different infection rate were observed in this study when compared to the previous investigation carried out in the same captive giant panda conservation center, *i.e.* CCRC. We propose that this might be due to the seasonal variation in the distribution of *Cryptosporidium* infection [2]. In our further studies, we will sample more fecal samples in different seasons of the year to determine the dynamics and full profiles of *Cryptosporidium* infection in giant pandas.

Regardless of the genotype*, Cryptosporidium* is a common parasite of wild mammals worldwide. Animals were exposed to *Cryptosporidium* after ingesting food or water that has been contaminated by infected feces [12]. Feng [13] reported that *Cryptosporidium* spp*.* infection rate in ungulates was 5.8 % (169/2896), 10.6 % (159/1506) in carnivores, 18.7 % (1937/10344) in wild rodents and 26.9 % (125/465) in primates [13]. In this study, the infection rate of *Cryptosporidium* observed in captive giant pandas (15.6 %) is higher than that observed in wild samples (0.5 %). This elevated captive infection rate may result from the relatively high density of captive housing and insufficient cleaned surfaces of the confined areas. High-density housing, to be more specific, is likely to not only increase the susceptibility of captive pandas to cryptosporidiosis, but also expose individuals to parasites that they would rarely encounter in the wild [14].

*C. andersoni* is the predominant species responsible for cryptosporidiosis in dairy and beef cattle [2]. The present observation revealed that adult pandas were more likely to be shedding *C. andersoni,* similar to previous studies that *C. andersoni* was more prevalent in yearling and adult cattle [15, 16]. In wild life, *C. andersoni* has been isolated from Bactrian camel (*Camelus bactrianus*), Bobak marmot (*Marmota bobac*), European wisent (*Bison bonasus*) [17], Mongolian gerbils (*Meriones unguiculatus*) [18] and lesser panda (*Ailurus fulgens*) [7]. Here, our results revealed the giant panda as a new host of *C. andersoni* and extended the range of host species known for this parasite. Although attention on zoonotic cryptosporidiosis has centered on other species such as *C. parvm, C. meleagridis*, *C. felis* and *C. canis* [2], previous studies showed that *C. andersoni* can infect humans under certain circumstances [19, 20]. Recently, *C. andersoni* was even identified as a novel predominant *Cryptosporidium* species in outpatients with diarrhea in China [21]. Due to the frequent contact of breeders, veterinarians and even tourists with captive giant pandas, the *C. andersoni* infections in captive giant pandas should be considered as a public health concern. The surveillance of the giant panda cryptosporidiosis is of great importance not only for veterinary medicine and researches but also for the public health concern.

### Conclusions

The present study revealed the existence of *C. andersoni* infection in giant panda for the first time, expanding the range of recognized host species for this parasite. Moreover, this molecular investigation provided useful information for more in depth research on the molecular epidemiology and control of *Cryptosporidium* infection in giant panda in the future.

## Abbreviations

18S rRNA: 18S ribosomal RNA
CRB: Chengdu Research Base of Giant Panda
CCRC: China Conservation and Research Centre for the Giant Panda
